# Case of Orbital Compartment Syndrome Post Cardiac Arrest

**DOI:** 10.7759/cureus.16514

**Published:** 2021-07-20

**Authors:** Roli Kushwaha, Johnathan Agil, Anthony Furiato, Amanda L Webb McAdams

**Affiliations:** 1 Emergency Medicine, Hospital Corporation of America West Florida Graduate Medical Education Consortium/Brandon Regional Hospital, University of South Florida Morsani College of Medicine, Brandon, USA; 2 Emergency Medicine, Brandon Regional Hospital, Brandon, USA; 3 Emergency Medicine, University of Central Florida College of Medicine, Orlando, USA

**Keywords:** ophthalmic emergency, lateral canthotomy, orbital compartment syndrome, non trauma ophthalmic case, emergency medicine procedures

## Abstract

Orbital compartment syndrome (OCS) is a rare ophthalmic surgical emergency in the setting of increased intraocular pressure (IOP) Irreversible vision loss can occur without immediate surgical treatment consisting of lateral cantholysis. We present a case of acute OCS discovered after cardiopulmonary resuscitation (CPR) with subsequent immediate lateral canthotomy and notable decrease in IOP. This in part demonstrates the importance of recognizing the clinical signs of OCS with or without trauma in the emergency department in addition with preparedness to perform a vision-saving procedure. Diagnosis is clinical and early recognition is essential. Index of suspicion for OCS in cardiac arrest without signs or history of trauma would typically be low, however, given the case presented, it was important for it to be excluded once the return of spontaneous circulation (ROSC) was achieved.

## Introduction

The orbit typically holds a volume of 30 mL containing globe, fat, extraocular muscles, vessels, nerves, lacrimal gland, and lacrimal sac held within fascial compartments [1-3]. The volume itself is confined by the orbital wall, orbital septum, and tarsal plate and intraocular pressure (IOP) is normally 8-21 mmHg [3-6]. Significant assault including but not limited to hemorrhage, abscess, tumor, orbital edema, or emphysema, or orbital cellulitis can lead to rapid rise in IOP >30 mmHg leading to ischemia and eventually irreversible vision loss [3,7,8]. Retinal ischemia for more than 90-120 minutes’ leads to high risk for permanent blindness and therefore, must be addressed immediately without any delay due to imaging [6,8]. Diagnosis of orbital compartment syndrome (OCS) is clinical and typically presents with the following: proptosis, ophthalmoplegia, relative afferent pupillary defect (RAPD), tense globe, tight eyelids in partially retracted position, elevated IOP, papilledema, optic atrophy, cherry red macula, venous congestion, or central retinal artery pulsation on fundoscopy [3,7,9-10]. Immediate surgical decompression lateral canthotomy and inferior canthotomy (LCIC) is the mainstay of treatment.

## Case presentation

**Figure 1:**
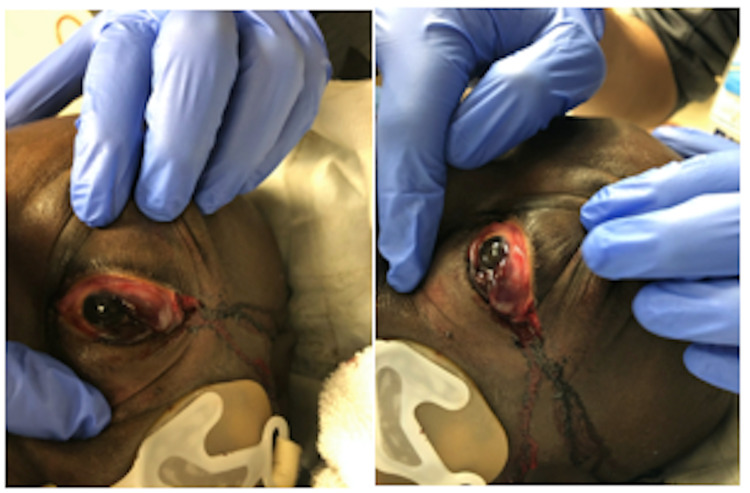
Post canthotomy (A) frontal view (B) lateral view

**Figure 2:**
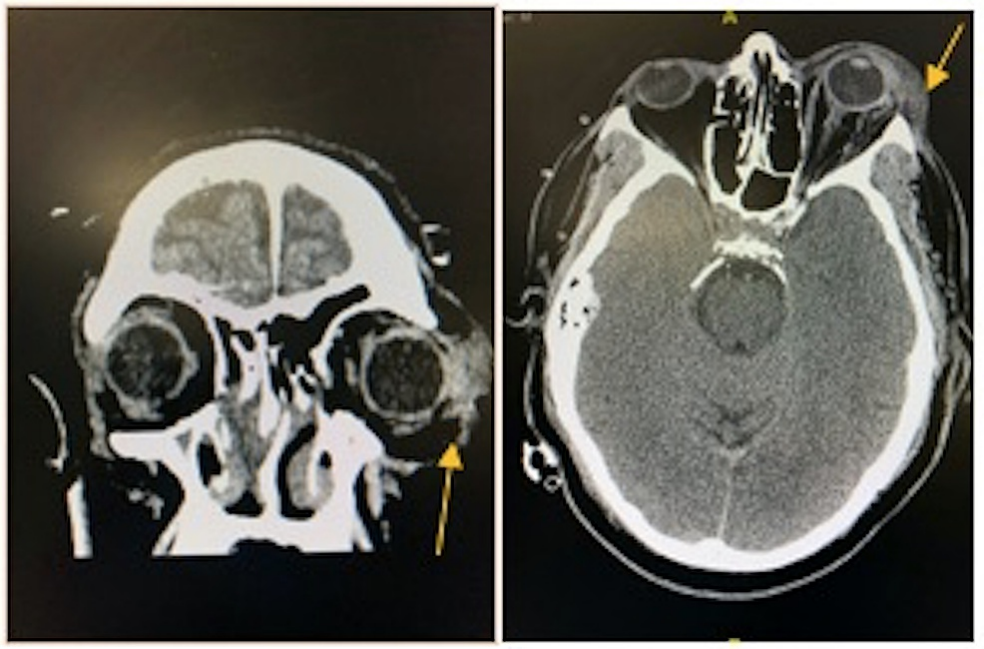
(A) CT brain coronal view (B) CT brain axial; yellow arrow pointing to soft tissue swelling left periorbital

A 57-year-old African-American male presented to the emergency department via emergency medicine service (EMS) with witnessed cardiac arrest. Patient reportedly was at work talking to a coworker when he collapsed and received immediate cardiopulmonary resuscitation (CPR) on scene. Patient was found in ventricular tachycardia arrest by EMS and received advanced cardiac life support (ACLS) protocol treatment with ROSC en route to the emergency department. Upon department arrival, the patient arrived with supraglottic device in place and remained unresponsive. No obvious signs of trauma were noted; however, the patient was noted to have leftward gaze with bilateral pupils responsive to light. The patient then went into ventricular tachycardia arrest upon being placed on department bed. CPR and ACLS protocol were initiated and continued for approximately three rounds when ROSC was achieved. Epinephrine drip was started and definitive airway was established. Soon after, the patient was noted to have spontaneous movements with opening eyes and was placed on Diprivan drip for sedation. While preparing for central line placement to the right internal jugular, it was noted that the patient’s left eye became significantly proptotic and chemotic appearing without pupil reactivity to light. IOPs were then immediately measured with tonometer device demonstrating pressure of 35 mmHg to the left eye and approximate pressure of 8 mmHg on the right. Decision to perform cantholysis was made given these findings. There was concern of retrobulbar hematoma likely in setting eye trauma upon patient collapsing prior to cardiac arrest. Using materials from laceration kit commonly found in the emergency department, hemostat pressure was applied to lateral canthus using Kelly clamp and then careful dissection of lateral canthus was made using tissue scissor. Upper and lower eyelids were retracted for better visibility of the superior and inferior crux, which were both cut given the extent of proptosis and chemosis (Figure 1). Post canthotomy pressure was measured at 11 mmHg and the left pupil was noted to be reactive. After surgical decompression, the patient underwent CT imaging of the brain which demonstrated soft tissue swelling of the left orbital region without evidence of retro bulbar hematoma (Figure 2). The patient was admitted to the medical intensive care unit for stabilization and later transferred to a tertiary care center for further ophthalmologic evaluation.

## Discussion

In this case, the patient presented in cardiac arrest without signs of trauma. On initial evaluation, the physical exam showed an unresponsive patient, supraglottic airway in place, and PERRLA (pupils equal, round, and reactive to light and accommodation) bilaterally and leftward gaze. There were no findings or portion of the history of present illness that would have led us to include OCS within the differentials. Incidence of OCS is 0.88% for the average patient population [11]. It is not routinely evaluated for post-cardiac arrest/ROSC due to the rare incidence. Post traumatic or postsurgical retrobulbar hemorrhages are typically the leading causes of OCS. However, prolonged hypoxemia with a capillary leak, which is more likely with our patient case and prolonged Valsalva maneuver, have been rare reported causes [11,6]. The primary goal for our patient was ROSC and stabilization. After achieving ROSC, the patient was prepped for central line placement during which time, physical exam findings of proptosis and chemosis without pupil reactivity to light were discovered. Although a thorough physical exam was performed earlier, there were no signs of OCS up until achieving ROSC and continuing stabilization. There would have been inevitable retinal ischemia if the physical findings stated above were not acted upon immediately. This further proves that timely orbital decompression is crucial within two hours of arrival [2]. This window can be even smaller for a vision-saving procedure. Prompt recognition and action were taken in this case. Markers of efficacy of the cantholysis demonstrated on our patient were improvement of proptosis, disappearance of afferent pupillary defect, and reduction of IOP to 11 mmHg [3].

Prompt action regardless of the mechanism was taken based on our physical exam findings. If globe rupture is unlikely, it is reasonable to delay imaging and opt to perform the procedure and preserve vision [8]. This case is a reminder that it is crucial to identify signs of OCS in any possible setting and preparedness of the emergency medicine physician to perform LCIC.

## Conclusions

This case presentation demonstrates the importance of prompt recognition of OCS and preparedness to perform lateral canthotomy in the emergency department. Although OCS may be extremely rare post-cardiac arrest and ROSC, routine repeat thorough physical exams are crucial in identifying OCS and performing lateral canthotomy in a timely fashion.
